# The Anti-Inflammatory Effects of Angiogenin in an Endotoxin Induced Uveitis in Rats

**DOI:** 10.3390/ijms21020413

**Published:** 2020-01-09

**Authors:** Jihae Park, Jee Taek Kim, Soo Jin Lee, Jae Chan Kim

**Affiliations:** 1 Department of Ophthalmology, Cheil Eye Research Institute, Cheil Eye Hospital, Daegu 803-2, Korea; jihae.park85@gmail.com (J.P.); tnwls21@naver.com (S.J.L.); 2 Department of Ophthalmology, College of Medicine, Chung-Ang University Hospital, Seoul 224-1, Korea; jeetaek-kim@hanmail.net

**Keywords:** angiogenin, uveitis, EIU rat model

## Abstract

Angiogenin (ANG) is involved in the innate immune system and inflammatory disease. The aim of this study is to evaluate the anti-inflammatory effects of ANG in an endotoxin induced uveitis (EIU) rat model and the pathways involved. EIU rats were treated with balanced salt solution (BSS), a non-functional mutant ANG (mANG), or wild-type ANG (ANG). The integrity of the blood-aqueous barrier was evaluated by the infiltrating cell and protein concentrations in aqueous humor. Histopathology, Western blot, and real-time qRT-PCR of aqueous humor and ocular tissue were performed to analyze inflammatory cytokines and transcription factors. EIU treated with ANG had decreased inflammatory cells and protein concentrations in the anterior chamber. Compared to BSS and mANG, ANG treatment showed reduced expression of IL-1β, IL-8, TNF-α, and Myd88, while the expression of IL-4 and IL-10 was increased. Western blot of ANG treatment showed decreased expression of IL-6, inducible nitric oxide synthase (iNOS), IL-1β, TNF-α, and phosphorylated NF-κB and increased expression of IL-10. In conclusion, ANG seems to reduce effectively immune mediated inflammation in the EIU rat model by reducing the expression of proinflammatory cytokines, while increasing the expression of anti-inflammatory cytokines through pathways related to NF-κB. Therefore, ANG shows potential for effectively suppressing immune-inflammatory responses in vivo.

## 1. Introduction

Ocular inflammation is one of the main causes of blindness and visual disturbance [1,2]. Uveitis is a sight threatening disease that results from acute, recurrent, or chronic inflammation of the uvea [1] and is involved with the immune system [3,4]. Despite aggressive treatment, chronic recurrence can be detrimental and cause irreversible visual impairment and blindness. It accounts for 10–15% of all blindness worldwide [5,6,7]. About 50% of the cases are associated with chronic systemic inflammatory diseases [8] such as rheumatoid arthritis [9], systemic lupus erythematosus [10], polyarteritis nodosa, relapsing polychondritis, Wegner’s granulomatosis, scleroderma, Bechet’s disease [11], and ankylosing spondylitis [12,13,14]. Endotoxin induced uveitis (EIU), a well established animal model for human acute anterior uveitis, is induced by lipopolysaccharide (LPS), which is released from Gram-negative bacteria [1,12]. LPS is specifically recognized by toll-like receptor 4 (TLR4) [4], a pattern recognition receptor of the innate immune system on the surface of macrophages [3,5,15]. When LPS binds to TLR4, it activates TLR4 and signals through the common adaptor protein, myeloid differentiation factor 88 (Myd88) [16], subsequently producing numerous proinflammatory cytokines [17], like tumor necrosis factor alpha (TNF-α) and interleukin-6 (IL-6), through nuclear translocation of nuclear factor-κB (NF-κB) [5,18]. EIU is portrayed by iris vasodilation and vascular alterations in the ciliary body, leading to augmented vascular permeability and blood-aqueous barrier breakdown [12,19]. Disruption of the blood-ocular barrier is characterized by infiltration of aqueous humor with inflammatory cells and rapid increase in total protein content [13,15,20,21,22]. The aim of treatment for chronic uveitis is long term control of inflammation. The current mainstay treatment for uveitis is topical or systemic steroids, but their long term use may have serious ocular and systemic side effects [4,23]. For refractory uveitis where high dose steroids are not effective, immunomodulating drugs are also used, but high concentrations may cause serious adverse effects [4,13,24]. Thus, more effective therapy is needed.

Angiogenin (ANG), also known as ribonuclease 5, is a 14.4 kDa, single chain protein containing 123 amino acids and is a natural component of tears [25]. It binds ribosomal DNA after being translocated into the nucleus and stimulates ribosomal RNA transcription for angiogenesis and cell proliferation [26]. As a stimulating factor for cell growth and angiogenesis [27], it is normally expressed in various human tissues [28,29,30,31,32]. Several studies have reported its association with innate immunity and its bactericidal effects [28,29]. The mRNA expression and protein concentrations of serum ANG are reportedly increased in inflammatory bowel disease [30] and dry eye disease [32], both of which are diseases of inflammation. Laurie et al. [32] have reported on the positive correlation between ANG concentration and severity of dry eye syndrome. However, it is unclear whether the purpose of this increase is to inhibit or aggravate inflammatory reactions. The data suggest that ANG could have both roles. Our current understanding of its exact role is still unclear, and its involvement in inflammation and immunity in various parts of the body from current research only shows a fragment of its true interactions.

Recently, we reported [33,34] that ANG has anti-inflammatory effects on the ocular surface through inhibiting TANK-binding kinase 1 (TBK1) expression and suppressing intranuclear transport of NF-κB in human corneal fibroblasts (HCFs). Our previous study showed that ANG decreased the proinflammatory cytokines IL-1β, -6, and -8, while increasing the anti-inflammatory cytokines IL-4 and -10 in corneal inflammation induced by TNF-α or LPS. To show that this effect could also be simulated in the uvea in vivo, we conducted a similar study on EIU. The aim of this present study was to evaluate the in vivo anti-inflammatory activity of ANG in parts of the ocular tissue other than the cornea. First, we attempted to verify whether ANG has anti-inflammatory effects in an EIU model. Second, we tried to clarify the mechanism behind its activity by studying the changes it induces in nuclear translocation of NF-κB and the involved cytokines. In this study, we particularly focused on the contribution of the TLR4/Myd88 pathway for activation of NF-κB.

## 2. Results

### 2.1. ANG Reduced Infiltrating Inflammatory Cells, Protein Content, and Proinflammatory Cytokines in the Anterior Chamber

**Figure 1:**
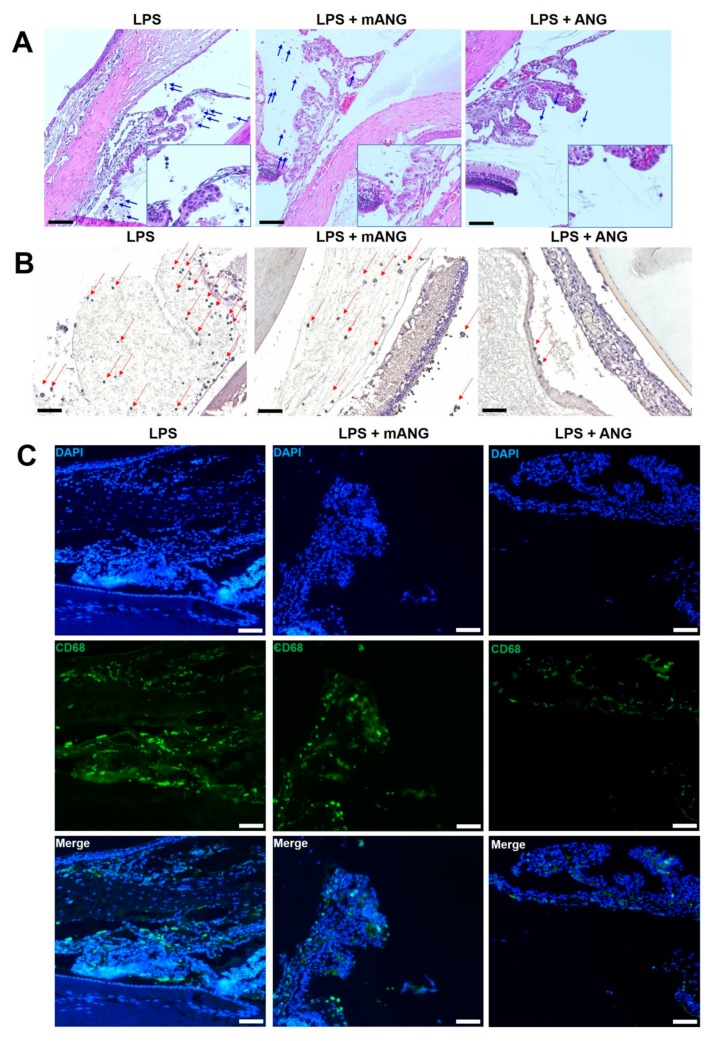
Histopathological evaluation of EIU in rats. (**A**) The eyes were cryosectioned and were stained with H&E. Representative sections are shown. Arrows show the infiltration of inflammatory cells (leukocytes) in the anterior chamber. Accumulation of fibrinous membrane is shown in both LPS and LPS + mANG eyes. (**B**) Immunohistochemical staining on frozen sections of the ciliary bodies. Arrows indicate the expression of macrophage marker CD11B. (**C**) The expression of CD68 was determined by immunofluorescent localization (green). Fluorescent staining of the nuclei of fixed cells is shown as the control in 4′,6-diamidino-2-phenylindole (DAPI) (blue). The same sections were localized for CD68 in all treatment groups, and the two different stains were merged to show the relative change in expression. All scale bars = 200 μm (*n* = 5).

Inflammation in the anterior chamber was assessed by histological analysis. LPS injection induced intense accumulation of infiltrating inflammatory cells and fibrinous membrane in the anterior chamber of the EIU eyes. Treatment with mANG had little or no effect in decreasing inflammation, while ANG treatment showed a substantial decrease in cells and fibrinous membrane in the anterior chamber. Representative histological sections are shown in Figure 1A. LPS(-) normal control eyes showed no evidence of infiltrating cells or fibrinous membrane formation. Immunohistochemical studies on frozen sections of the ciliary bodies were also performed. Tissue sections stained for localization of macrophage markers CD11B and CD68 showed intense staining in LPS induced eyes (Figure 1B,C). While CD11B- and CD68-positive cells were slightly decreased in mANG treated eyes, significant decreases in both markers were detected in ANG treated eyes.

**Figure 2:**
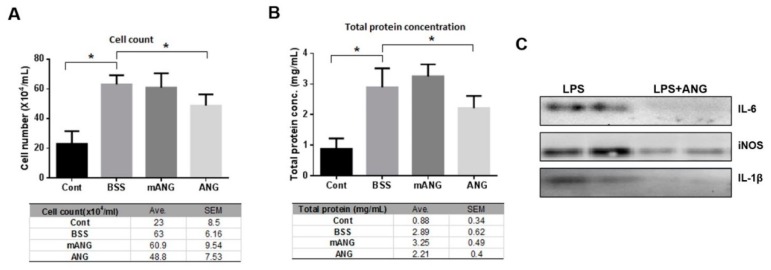
ANG attenuates inflammation in EIU. ANG attenuates inflammation in EIU. (**A**) Quantification of infiltrating cells in the anterior chamber. LPS induced EIU eyes showed significantly increased cell count compared to the normal LPS control. ANG treated eyes contained significantly lower numbers of infiltrating leukocytes in the anterior chamber (48.8 ± 7.53) compared to BSS or mANG treated eyes (63 ± 6.16 or 60.9 ± 9.54; both *p* < 0.05). (**B**) Quantification of infiltrating protein concentration in the anterior chamber. The total protein content was significantly lower in ANG treated eyes compared with LPS + BSS control eyes (2.21 ± 0.4 and 2.89 ± 0.62, respectively; both *p* < 0.05), indicating better preservation of the blood-aqueous barrier (* *p* < 0.05, Avg = mean). (**C**) Western blot analyses of proinflammatory cytokines and enzyme in the anterior chamber of LPS induced eyes. ANG treatment decreased all the expressions of IL-6, iNOS, and IL-1β (*n* = 5 rats per group).

To assess the severity of EIU, changes in the total level of inflammatory cells and protein concentration in the anterior chamber were also analyzed. The inflammatory cell count in the anterior chamber after LPS injection was 23 ± 8.5 for LPS(-) control. In LPS + BSS and LPS + mANG treated eyes, the cell counts were 63 ± 6.16 and 60.9 ± 9.54, respectively, showing induction of severe inflammation following LPS injection. The eyes treated with ANG showed significantly lower inflammatory cell counts of 48.8 ± 7.53 in the aqueous humor at the same time point, indicating effective local immunosuppression (Figure 2A).

The aqueous total protein content, which represents the integrity of the blood-aqueous barrier, showed a similar trend to total cell count. Compared to the LPS(-) control, a significant increase in total protein of the anterior chamber was observed in the LPS + BSS group, indicating disrupted blood-aqueous barrier integrity. No significant decrease was noted in the mANG treated group, but rather an increase was observed. In ANG treated eyes, total protein concentration was 2.21 ± 0.4, which was significantly lower than in the BSS treated group (2.89 ± 0.62), suggesting better preserved blood-aqueous barrier (Figure 2B). Western blot analysis of the aqueous humor showed that ANG application reduced the expression of proinflammatory cytokines IL-6, -1β, and proinflammatory enzyme iNOS compared to LPS + BSS (Figure 2C).

### 2.2. ANG Inhibits mRNA Expression of Proinflammatory Cytokines and Myd88 While Promoting mRNA Expression of Anti-Inflammatory Cytokines in Ocular Tissues

**Figure 3:**
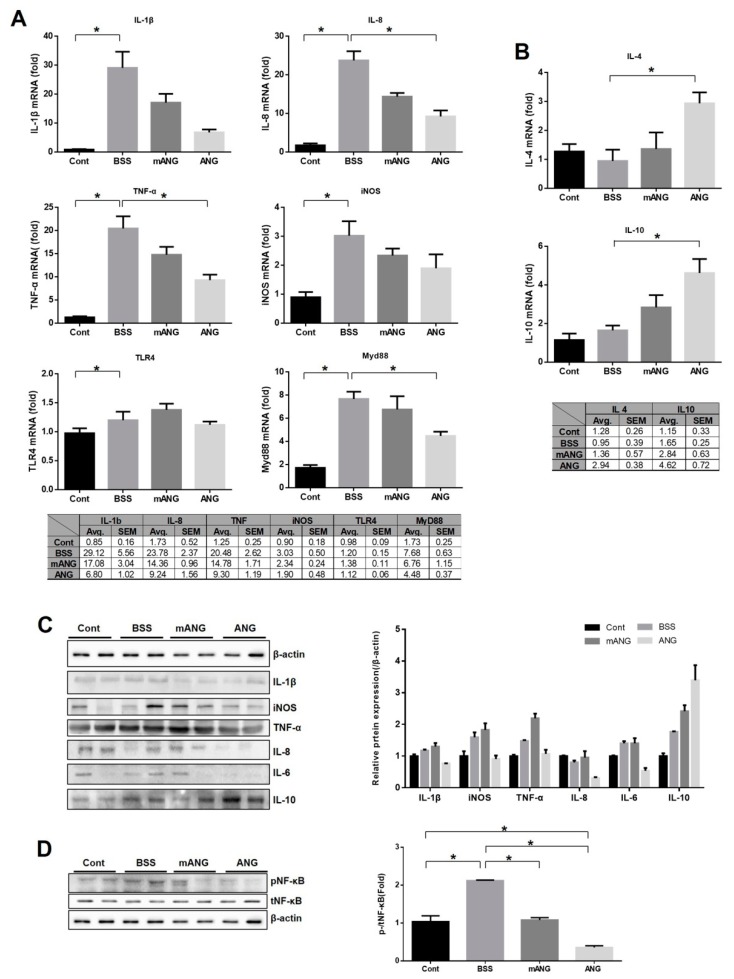
Real-time qRT-PCR analyses and Western blot of proinflammatory and anti-inflammatory cytokines, TLR4, and Myd88 in LPS induced rat eye tissue with ANG or mANG or without treatment. (**A**) The increased relative mRNA levels of IL-1β, IL-8, TNF-α, iNOS, and Myd88 were diminished by ANG treatment. However, TLR4 was not reduced upon ANG treatment. Treatment with mANG had no significant effect on reducing the mRNA expressions. (**B**) The relative mRNA levels of anti-inflammatory cytokines IL-4 and IL-10 were increased by ANG treatment. Treatment with mANG had no significant effect on increasing them; values represent the mean ± standard error (* *p* < 0.05, Avg = mean). (**C**) Western blot analyses showed ANG treatment reduced iNOS, TNF-α, and IL-8 protein expressions in LPS induced EIU eyes. (**D**) After ANG treatment, nuclear translocation of NF-κB induced by LPS was decreased as seen by reduced pNF-κB expression and decreased p-/tNF-κB. The cytosolic NF-κB was not affected by either ANG or mANG (*n* = 5 rats per group).

Real-time qRT-PCR was performed to determine the cytokine and transcription factor responses to LPS induction and ANG treatment in ocular tissue (Figure 3A,B). The mRNA from proinflammatory and anti-inflammatory cytokines was assessed. Significant increases in mRNA expression of all proinflammatory cytokines (IL-1β, IL-8, and TNF-α) and iNOS examined were observed with LPS injection. They all showed downregulation of mRNA expression with ANG treatment. Especially for IL-8 and TNF-α, ANG treatment led to significant reduction (*p* = 0.019 for both). However, EIU treated with mANG (LPS + mANG) exhibited no significant change from the LPS + BSS group. The mRNA expression of anti-inflammatory cytokines IL-4 and IL-10 was not significantly different between the LPS(-) control and the LPS + BSS group. In the ANG treated group, a significant increase in mRNA expression of both IL-4 and IL-10 was observed (*p* = 0.013 and *p* = 0.046, respectively), while mANG treatment did not induce any significant change compared to the LPS + BSS group.

To determine whether ANG can reduce the inflammatory response through the TLR4/Myd88 pathway, TLR4 and Myd88 were both analyzed with real-time qRT-PCR (Figure 3A). LPS induction significantly increased the mRNA expression of TLR4 and Myd88, but significant downregulation was noted after ANG treatment in Myd88 only. ANG treatment did not affect the mRNA expression of TLR4. No significant change in either TLR4 or Myd88 was induced by mANG treatment. It is interesting to note that, while ANG treatment resulted in significantly reduced expression of Myd88, it did not meaningfully affect TLR4, although both are key molecules in the NF-κB pathway.

Western blot of inflammatory factors revealed that ANG significantly reduces the expression of iNOS, TNF-α, pNF-κB, IL-6, and IL-8 compared to BSS or mANG groups. While IL-1β was not significantly affected by ANG, the anti-inflammatory cytokine IL-10 was significantly increased upon ANG treatment compared to BSS or mANG treatment (Figure 3C). Most interestingly, the nuclear translocation of NF-κB, as indicated by phosphorylated NF-κB (pNF-κB), induced by LPS was decreased after ANG treatment. This was seen by the reduced expression of pNF-κB in ANG group. The total NF-κB (tNF-κB), which is the inactive cytosolic form, was not affected by ANG or mANG. It is important to see the changes in the ratio of pNF-κB expression compared to tNF-κB. A significant decrease of pNF-κB/tNF-κB was noted after ANG treatment (Figure 3D).

### 2.3. ANG Inhibits Nuclear Translocation of NF-κB in the Ciliary Body

**Figure 4:**
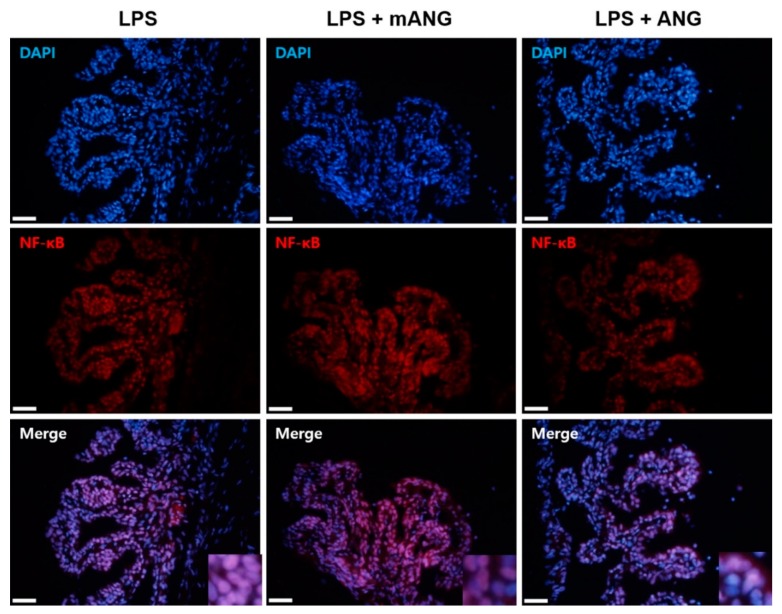
Immunofluorescent localization of NF-κB. The presence of NF-κB in the nuclei and cytoplasm of ciliary bodies of EIU eyes is shown in red. Fluorescent staining of the nuclei of fixed cells is shown as the control (DAPI) (blue). Merge images show the representative localization of NF-κB (purple). Note the relatively higher ratio of NF-κB in the nucleus in LPS than in ANG treated eyes. Scale bars = 200 μm (*n* = 5).

After LPS induction, the eyes were treated with mANG or ANG to examine whether ANG inhibits nuclear translocation of NF-κB. The sections of ciliary bodies were subjected to immunofluorescent localization of NF-κB. Immunohistochemical staining of NF-κB in the nucleus showed that treatment with LPS induced translocation of NF-κB into the nucleus. However, the presence of ANG inhibited translocation of NF-κB. Treatment with mANG had little influence on the expression of NF-κB in nucleus, as the result was similar to the LPS induction alone (Figure 4).

## 3. Discussion

In this preliminary study, we investigated the immune-regulatory effects of ANG in an animal model of acute uveitis. To explore the therapeutic effect of ANG in vivo, ANG, mANG, and BSS were administered topically in EIU, the most widely used model of human acute uveitis [35]. LPS injection produced successful induction of ocular inflammation in study rats through an increase in total cell count and leakage of protein in the anterior chamber. When the EIU rats were treated with mANG, little therapeutic effect was shown. However, with ANG treatment, many of the cells and proteins were reduced. ANG also suppressed the expressions of proinflammatory cytokines, such as IL-1β, IL-8, and TNF-α, and proinflammatory enzyme iNOS [36], while upregulating the expression of anti-inflammatory cytokines, like IL-4 and IL-10 [37,38], in EIU. These anti-inflammatory effects are associated with the downregulation of TLR4′s adaptor protein Myd88, required for TLR intracellular signaling [16]. Finally, intranuclear transport of NF-κB, indicated by the phosphorylated form of NF-κB (pNF-κB), was reduced (an important step for deactivating inflammation) [3,39,40,41]. These results provide evidence of the possible role of ANG in attenuating inflammation in EIU through inhibiting the steps involved in nuclear translocation of NF-κB.

ANG has been recognized to play an important role in angiogenesis and cell proliferation [42], migration, and differentiation [43,44]. Although most recent studies have focused on its potential as an anti-angiogenin based cure for cancer [45,46], it is also known for its antimicrobial activity and association with diverse inflammatory diseases and innate immunity [43]. Until recently, no evidence existed in the literature to elucidate whether its association with inflammation was to aggravate or to tame it. Very little mechanistic evidence of ANG existed to suggest its true association. In our previous studies [33,34], we showed that ANG has anti-inflammatory functions in inflamed HCFs through reduction of TNF-α and inhibition of nuclear transport of NF-κB. This was a novel finding of a previously undiscovered mechanism of ANG in inflammatory conditions. However, since it was confined to in vitro tests, it was hard to extend its effect in vivo. To understand the interactions of ANG fully, it is important to know how it interacts in animal models. The most meaningful finding of our current study was that ANG attenuated inflammation by inhibiting the NF-κB pathway in vivo, which was in accordance with the results from our previous in vitro studies.

TNF-α recognition by the TNF receptor (TNFR) and the activation of TLR4 by LPS are important steps in innate immune responses to inflammation [39,47,48]. Inflammation triggered by LPS and its main receptor TLR4 signals through Myd88, which signals through a cascade of intracellular events, leading to translocation of NF-κB into the nucleus and transcription of genes for proinflammatory cytokines [16,39,48,49,50]. The LPS/TLR4 interactions have been extensively studied [39,51]; however, we are still refining our understanding of the ensuing intracellular interactions leading to an inflammatory response. In this study, we demonstrated that the anti-inflammatory effects of ANG may be associated with direct interactions with Myd88 and/or direct translocation of ANG into the nucleus. Mutant-type ANG was reconstructed to inhibit its nuclear translocation [52]; in our study, mANG had little effect on reducing inflammatory conditions in EIU. Furthermore, the anti-inflammatory effects of ANG were barely associated with changes in TLR4, but directly affected the expression of Myd88. It is possible that the activation of NF-κB nuclear translocation is induced by intracellular signaling activated by direct interactions between ANG and Myd88. This was substantiated by the results of other studies [53] documenting that ANG could be internalized into the cytoplasm and be translocated into the nucleus without cell surface receptors, including TLRs. Therefore, direct internalization of ANG through the Myd88 pathway may be a possible mechanism by which it induces immune modulator effects.

**Figure 5:**
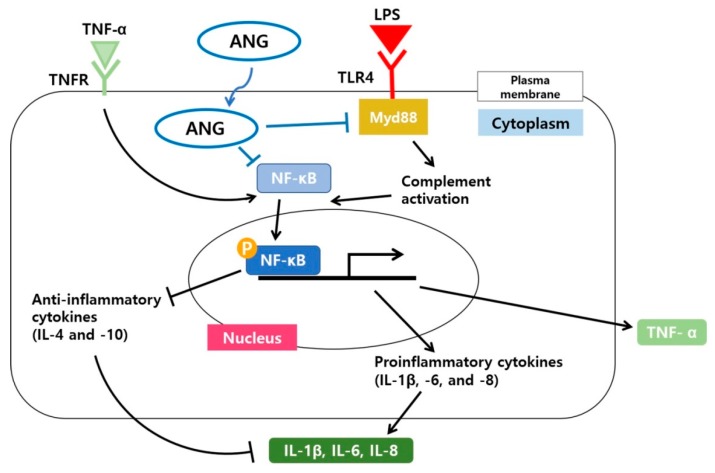
Schematic model illustrating the signaling pathway by which ANG reduces the inflammatory responses involving Myd88 and the nuclear translocation of NF-κB in LPS induced EIU. A possible direct internalization of ANG and the subsequent downregulation of Myd88 and nuclear translocation of NF-κB result in the reduced mRNA expression of proinflammatory cytokines (IL-1β, -6, and -8) and increased mRNA expression of anti-inflammatory cytokines (IL-4 and -10). Overall, the cascade underlying the effect of ANG results in suppression of inflammation.

NF-κB is a crucial heterodimer transcription factor in cell proliferation, inflammation, and immune responses [54,55]. When inactive, it is present in the cytosol of most cells and tissue types [56]. Phosphorylation of nuclear p65, one of the five components that form NF-κB, is an essential modification for the DNA binding activity of NF-κB [55,57]. In the NF-κB signaling pathway, p65 is largely involved in inflammatory responses [58]. Topical treatment with ANG caused diminished expression of pNF-κB, but not NF-κB, suggesting that the anti-inflammatory effects of ANG are associated with direct translocation of ANG into the nucleus, aiding in the downregulation of NF-κB activity. A schematic diagram illustrating the anti-inflammatory signaling pathways induced by ANG in LPS induced EIU is shown in Figure 5.

Our study has several limitations. First, the number of experimental animals was not large enough, and we did not repeat the entire experiment to validate its credibility. We also did not assess the expression level of ANG in control or EIU eyes. To understand the role of ANG in uveitis fully, these additional experiments should be performed to discover how the expression of ANG is affected by uveitis. Second, there are discrepancies between protein expression and mRNA expression in some cytokines. The Western blot data did not show LPS induced upregulation of IL-1β, -6, and -8. Instead, IL-10 appeared to be upregulated by LPS. However, the poor correlation between mRNA and the corresponding protein level has been well documented [59]. The frequently observed discrepancy is largely influenced by various regulators, such as transcript and protein products, at different levels along with other technical factors [60,61]. Third, the lack of involvement of TLR4 may be a meaningful novel finding or a result of an undetected error in the experiment. Again, additional studies are needed to confirm such conclusions.

Despite these limitations, our current study extends our previous results by demonstrating that, overall, ANG attenuates inflammation in uveitis. Topical application of ANG showed immune suppressive potential and provided insight into the anti-inflammatory role of ANG in ocular inflammation in vivo. Moreover, its mechanism may be associated with the Myd88 and NF-κB pathway. In conclusion, ANG could be an effective and safe new therapy for inflammation in uveitis and other inflammatory diseases of the eye. However, further investigation is required to elucidate the exact mechanisms of ANG and to validate our hypothesis prior to clinical application of ANG in ocular inflammation.

## 4. Materials and Methods

### 4.1. Study Approval and Material Preparation

All experiments were performed in accordance with The Association for Research in Vision and Ophthalmology (ARVO) statement on the Use of Animals in Ophthalmic and Vision Research and were approved by the local animal care committee (Approval code: No.14-00015; Approval date 2014.03.30.). All animals were kept in the Chung-Ang University Animal Care Center.

Mutant-type rat ANG (mANG) and wild-type rat ANG (ANG) were obtained from the Department of Biochemistry at Chungbuk National University (Cheongju, South Korea). The identity of the purified ANG was confirmed by Western blotting with ANG specific antibodies as described previously [62]. Mutant-type ANG was generated to inhibit internalization and nuclear translocation through structural changes in ANG heparin binding sites via double mutations R31A/R32S and K50L/K54Q of rat ANG [52].

### 4.2. Induction of Anterior Uveitis (EIU) in Rats and Topical Treatment with ANG Eye Drops

Six-week-old male Sprague–Dawley rats weighing approximately 150 to 160 g were used. Anterior uveitis was induced by a single footpad injection of 150 μg lipopolysaccharide (LPS; Sigma-Aldrich, Saint-Quentin, France) dissolved in phosphate buffered saline (100 μL PBS, pH 7.4). This EIU model was treated with mANG eye drops, ANG eye drops (mANG and ANG both dissolved in the same phosphate buffered saline mentioned above), or balanced salt solution (BSS; Alcon Laboratories, Fort Worth, TX, USA) eye drops (*n* = 5 rats per group) immediately after LPS injection. Eye drops were applied four times a day for three days at doses of 100 μg/mL. Rats in an LPS-negative control (LPS(-) control) group were injected with vehicle (BSS) instead of LPS and were not treated. Rats were euthanized three days after treatment. Immediately after euthanizing the animals, aqueous humor or ocular tissue were collected from both eyes. The treatments and number of rats used are shown in Table 1.

### 4.3. Cell Counting and Quantification of Protein Concentration in Aqueous Humor of EIU Rats

Aqueous humor was obtained from both eyes by anterior chamber (AC) puncture with a 30 gauge needle (*n* = 5 rats per group). Aqueous humor samples were kept on ice until analysis. From the total 80 μL of aqueous humor obtained from the AC, 10 μL were used for cell counting, and 70 μL were used for Western blot. Cell counting and total protein quantification were performed within 1 h of sample collection. For cell counting, aqueous humor was suspended in an equal amount of 0.4% trypan blue solution for counting with a hemocytometer under a light microscope (Olympus Optical Ltd., London, U.K.). Total protein concentration was measured via the Pierce BCA protein assay kit (Thermo Fisher Scientific, Waltham, Massachusetts, USA) (*n* = 5 rats per group) according to the manufacturer’s protocol.

### 4.4. Histopathological Evaluation of EIU Rats

Eyes were fixed in 4% paraformaldehyde overnight and then were cryoprotected with 30% sucrose overnight and embedded in optimal cutting temperature (OCT) compound (Tissue-Tek^®^ OCT Compound; Sakura Finetek USA, Inc., Torrance, CA, USA) on dry ice at −80 °C. Frozen OCT compound embedded sections were cut at 8 μm thickness, placed on silane coated microscope slides, and stained with hematoxylin and eosin (H&E). Frozen sections were determined by immunohistochemistry using anti-CD11b (rabbit IgG, diluted to 1:100 in PBS; Thermo Fisher Scientific, Waltham, Massachusetts, USA) overnight at 4 °C. The primary antibodies were detected by an ABC kit (Vectastain Elite; Vector Laboratories, Burlingame, CA, USA) and counterstaining with hematoxylin and observed with light microscopy (Leica DM750; Leica Microsystems Ltd., Wetzlar, Germany). For immunofluorescence, frozen sections were permeabilized by incubation with 0.3% Triton X-100 for 15 min at room temperature and then incubated with anti-NF-κB (rabbit IgG, diluted to 1:50 in PBS; Bioworld Technology, Inc., St. Louis Park, MN, USA) and anti-CD68B (mouse IgG1, diluted to 1:80 in PBS; Thermo Fisher Scientific, Waltham, Massachusetts, USA) overnight at 4 °C. Sections were incubated with secondary antibody (anti-rabbit IgG Alexa Fluor 594 and anti-mouse IgG Alexa Fluor; Thermo Fisher Scientific, Waltham, Massachusetts, USA) for 1 h at room temperature. At each step, slides were washed three times (5 min each) with PBS. Cover slips were mounted on slides using Vectashield (Vector Laboratories, Burlingame, CA, USA) containing 40,6-diamidino-2-phenylindole (DAPI). The stained tissue was observed using an inverted microscope (IX71, Olympus Optical Ltd., London, U.K.).

### 4.5. Purification of Total RNA and Real-Time qRT-PCR from Ocular Tissue of EIU Rats

Eyes were removed and immediately frozen in liquid nitrogen. Frozen eyes were cut in two; one half was used for RNA isolation. After thawing the eyes, the correct volume of RNAiso Plus (Takara Bio Inc., Otsu, Japan) required for tissue homogenization was determined. The quantity and quality of RNA were determined using a NanoDrop ND-1000 spectrophotometer (ND-1000; Nano-Drop Technologies, Inc., Wilmington, DE, USA). Single strand complementary DNA (cDNA) was synthesized from 1 μg total RNA using cDNA synthesis kits (Takara Bio Inc. Otsu, Japan). Real-time qRT-PCR was conducted using a CFX96TM Real-Time PCR Detection System (Bio-Rad, Hercules, CA, USA) in a total of 20 μL containing 10 μL SYBR Premix Ex Taq (Takara Bio Inc. Otsu, Japan), diluted cDNA template and forward and reverse primers. Relative gene quantities were obtained using the comparative cycle threshold (Ct) method after normalization to a reference gene (glyceraldehyde-3-phosphate dehydrogenase [GAPDH]). The results of the real-time qRT-PCR analysis are presented as the average amount of each gene expressed relative to average reference gene expression. Primer sequences are listed in Table 2.

### 4.6. Western Blots of Eyes from EIU Rats

Eyes were removed, immediately frozen in liquid nitrogen, and stored at −80 °C. Frozen eyes were cut in two; one half was used for protein isolation. After thawing, eyes were placed on ice, and 200 μL protein extraction solution (PRO-PREP; iNtRON, Seongnam, South Korea) were used for tissue homogenization. Tissue lysates were separated on sodium dodecyl sulfate (SDS)-polyacrylamide gels and transferred to polyvinylidene fluoride membranes (PVDF; Merck Millipore, Billerica, MA, USA). Nonspecific antibody binding was blocked with 5% skim milk in TBS-T (50 mM Tris-HCl pH 7.5, 150 mM NaCl, and 0.1% Tween-20) for 1 h at room temperature. Primary antibodies against IL-1β, IL-6, IL-8, IL-10, inducible nitric oxide synthase (iNOS), NF-κB, phosphorylated NF-κB (pNF-κB), and β-actin were diluted in TBS-T containing 5% BSA (1:1000) before application to PVDF membranes and overnight incubation at 4 °C. Secondary antibodies diluted in TBS-T containing 5% skim milk (1:2000) were incubated for 1 h at room temperature. Specific antibody binding was visualized using enhanced chemiluminescence Western blotting detection kits (Pierce Biotechnology, Inc., Rockford, IL, USA). The value of each band was normalized to that of β-actin. Quantification of immunobands was performed using ImageJ software (LOCI; University of Wisconsin, Madison, WI, USA).

### 4.7. Statistical Analysis

ANOVA was used to analyze significant differences (SPSS 19.0; IBM Corporation, Chicago, IL, USA). Differences were considered statistically significant at *p* < 0.05. Data are expressed as the mean ± the standard error of the mean (SEM).

## 5. Conclusions

ANG seemed to reduce immune mediated inflammation in the EIU rat model effectively by reducing the expression of proinflammatory cytokines and enzyme, while increasing the expression of anti-inflammatory cytokines through pathways related to Myd88 and NF-κB. Therefore, ANG showed potential for effectively suppressing inflammatory responses in vivo.

## Figures and Tables

**Table 1:** Summary of the treatment groups.

Footpad Injection	Eye Drop	Treatment Group	Number of Animals
Saline	BSS	Cont	5
LPS	BSS	BSS	5
LPS	Mutant angiogenin	mANG	5
LPS	Wild angiogenin	ANG	5

**Table 2:** Sequences of PCR primers.

Gene	Forward Sequence (5′-3’)	Reverse Sequence (5’-3’)	Product Size (bp)
IL-1β	CACCTTCTTTTCCTTCATCTTTG	GTCGTTGCTTGTCTCTCCTTGTA	241
IL-4	GTCACTGACTGTAGAGAGCTATTG	CTGTCGTTACATCCGTGGATAC	107
IL-8	AGACAGTGGCAGGGATTCAC	GAGTGTGGCTATGACTTCGGT	95
IL-10	CTGCTATGTTGCCTGCTCTTA	GGGAAGTGGGTGCAGTTATT	86
TNF-α	GACCCTCACACTCAGATCATCTTC	TTGTCTTTGAGATCCATGCCATT	147
iNOS	TTGGGTCTTGTTAGCCTAGTC	TGTGCAGTCCCAGTGAGGAAC	262
TLR4	GGCATCATCTTCATTGTCCTTG	AGCATTGTCCTCCCACTCG	111
Myd88	AGAGTGGAGAGCAGTGTC	GGCAGTAGCAGATGAAGG	109
GAPDH	GCAAGGATACTGAGAGCAAG	GGATGGAATTGTGAGGGAGA	98
